# ﻿Unravelling the diversity of the genus *Afronurus* Lestage, 1924 (Ephemeroptera, Heptageniidae) in Thailand

**DOI:** 10.3897/zookeys.1176.105159

**Published:** 2023-08-22

**Authors:** Anuntaya Wongyam, Michel Sartori, Boonsatien Boonsoong

**Affiliations:** 1 Animal Systematics and Ecology Speciality Research Unit (ASESRU), Department of Zoology, Faculty of Science, Kasetsart University, Bangkok 10900, Thailand Kasetsart University Bangkok Thailand; 2 State Museum of Natural Sciences, Department of Zoology, Palais de Rumine, Place de la Riponne 6, CH-1005 Lausanne, Switzerland State Museum of Natural Sciences, Department of Zoology Lausanne Switzerland; 3 Department of Ecology and Evolution, University of Lausanne, Biophore, CH-1015 Lausanne, Switzerland University of Lausanne Lausanne Switzerland; 4 Biodiversity Center Kasetsart University (BDCKU), Bangkok 10900, Thailand Biodiversity Center Kasetsart University (BDCKU) Bangkok Thailand

**Keywords:** COI, mayfly, systematics

## Abstract

The genus *Afronurus* in Thailand is investigated using an integrative approach (morphology based, ootaxonomy and molecular data) for species delimitation. A total of four species of *Afronurus* was identified; *A.cervina* (Braasch & Soldán, 1984), *A.gilliesiana* (Braasch, 1990), *A.rainulfiana* (Braasch, 1990), and *A.rubromaculata* (You et al., 1981). The subimago of *A.gilliesiana* is described for the first time based on reared specimens. The egg structure of all four species is also described for the first time. Morphological and molecular data strongly support their species delimitation. The egg chorionic structure of the genus *Afronurus*, together with other morphological characters, is useful for species identification. A key to mature nymphs of the known species is provided.

## ﻿Introduction

Members of the family Heptageniidae are among the most abundant and common components of benthic communities in running waters, and many heptageniid species have been used as indicators of anthropogenic disturbance. This family is distributed mainly in the Holarctic, Oriental, and Afrotropical regions, with around 509 described species (Sartori and Brittain 2015). Heptageniidae from Thailand have previously been investigated (Braasch 1990; Sites et al. 2001; Sangpradub et al. 2002; Wang and McCafferty 2004; Webb and McCafferty 2008; Braasch and Boonsoong 2010; Boonsoong and Braasch 2013; Boonsoong et al. 2021), where ten genera and approximately 26 species have been recorded (Boonsoong 2022).

The genus *Afronurus* was established by Lestage (1924) from the African representatives assigned to the genus *Ecdyonurus*. Wang and McCafferty (2004) and Kluge (2004) synonymised the Asian genus, *Cinygmina* Kimmins, 1937, with *Afronurus*. The nymphs of the genus *Afronurus* can be distinguished from those of other genera by the presence of two rows of long, fine setae on the mid and hind tibiae, simple scattered setae on the ventral surface of the maxillae, and a slightly thickened anterior margin of the head capsule (Webb and McCafferty 2008). The genus *Afronurus* is the third largest heptageniid genus, with more than 65 known species from the Oriental (44 species), Afrotropical (15 species), and Palearctic (6 species) realms (Sartori and Brittain 2015; Yanai et al. 2017; Zhang et al. 2021). In Southeast Asia, many species are known only as nymphs, or described as adults (Braasch and Soldán 1984; Braasch 1990; Nguyen and Bae 2003; Braasch 2011; Braasch and Jacobus 2011).

The nymphs of the genus *Afronurus* are among the most common heptageniids encountered in Thai streams (Boonsoong and Braasch 2013). Six species of this genus have been reported in Thailand, namely *A.cervina* (Braasch & Soldán, 1984), *A.dama* (Braasch & Soldán, 1987), *A.gilliesiana* (Braasch, 1990), *A.namnaoensis* Braasch & Boonsoong, 2010, *A.rainulfiana* (Braasch, 1990), and *A.rubromaculata* (You et al., 1981) (Boonsoong and Braasch 2013). However, information is lacking regarding the association between the nymphal and imaginal stages and the genetic identity of known *Afronurus* species in Thailand. The aim of this paper is to study the systematics of the genus *Afronurus* in Thailand using morphology-based and mitochondrial COI analyses.

## ﻿Materials and methods

### ﻿Morphological observation

*Afronurus* nymphs were collected from different microhabitats in streams in Thailand. The nymphs were ﬁxed and kept in absolute ethanol. Some nymphs with dark wing pads were selected for rearing to the adult stage for association. Photographs were taken using a Nikon SMZ800 stereoscopic microscope. The eggs of *Afronurus* were dried in a critical point drier (Polaron Range CPD7501) and coated with gold (Polaron Range SC7620) for examination of the chorionic structures by scanning electron microscopy (Quanta 450). Voucher specimens are deposited in the Aquatic Insects Collection of the Zoological Museum Kasetsart University (**ZMKU**), Bangkok, Thailand.

### ﻿Molecular analysis

Total genomic DNA was extracted from the legs on one side of a nymph using a Genomic DNA Purification Kit (NucleoSpin, Macherey-Nagel, Germany) following the manufacturer’s protocol. The COI gene was amplified using primers LCO1490 (5’-GGT CAA CAA ATC ATA AAG ATA TTG G-3’) and HCO2198 (5’-TAA ACT TCA GGG TGA CCA AAA AAT CA-3’), designed by Folmer et al. (1994). The polymerase chain reaction (PCR) conditions as were follows: a 50 μL final reaction volume containing 25 μL of PCR Master mix Solution (i-Taq), 1 μL (10 μm) of each primer, 2 μL of total DNA and 21 μL of nuclease-free sterile water, using the PCR conditions described by Gattolliat et al. (2015). Amplification cycles were as follows: 5 min at 94 °C, then 30 s at 94 °C, 30 s at 48 °C and 60 s at 72 °C (40 cycles), and a final elongation step at 72 °C for 10 min. The PCR products were purified and sequenced by Macrogen, Inc., Korea.

Sequence alignment and editing were performed using MEGA X (Kumar et al. 2018). The best-fit models were selected based on MEGA X. The General Time Reversible Model and Gamma distributed with invariant sites (GTR+G+I) were used for the MrBayes analysis, v. 3.1.2 (Huelsenbeck and Ronquist 2001). Analyses of four MCMC chains run for one million generations with trees sampled every 100 generations were used for BI. The stationary nucleotide frequencies, the alpha shape parameter of the gamma distribution, the relative rate of substitution and the proportion of invariant sites were unlinked across partitions. The first 25% were discarded as a burn-in after visually verifying that likelihood curves had flattened out and that the independent runs converged using Tracer 1.5 (Drummond and Rambaut 2007). Nucleotide sequences obtained in this study are deposited in the GenBank database (Table 1). Other analysed sequences from *Afronurus* and related genera, obtained from the Barcode of Life Data System (BOLD), were *A.mnong* (OP347112.1) and *A.meo* (OP347111.1) from Vietnam, *A.hyalinus* (LC377346.1) and *A.rubromaculata* (MK642294.1) from China and *Anaposzebratus* (HG935069.1) as the outgroup. We followed all guidelines of the Animal Ethics Committee of Kasetsart University (approval no. ACKU63-SCI-005 and ACKU66-SCI-017) for collecting the mayfly specimens.

**Table 1.** List of sequences of four Thai *Afronurus* species at each sampling site.

Species	Code	Collection locality	Date	GenBank accession number
*A.cervina* (Braasch & Soldán, 1984)	SP04LE	Loei	17 Dec 2018	OP729860
	SP04LE2	Loei	17 Dec 2018	OP729859
	SP04LE3	Loei	17 Dec 2018	OP729861
	SP04LE4	Loei	17 Dec 2018	OP729862
	SP01KN	Kanchanaburi	31 Jan 2019	OP729856
	SP01KN2	Kanchanaburi	11 Apr 2015	OP729857
	SP01KN3	Kanchanaburi	31 Jan 2019	OP729852
	SP01KN4	Kanchanaburi	31 Jan 2019	OP729858
	SP01PC	Petchburi	25 Feb 2018	OP729850
	SP01PK	Prachuap Khiri Khan	19 Apr 2019	OP729848
	SP01PK2	Prachuap Khiri Khan	19 Apr 2019	OP729853
	SP01PK3	Prachuap Khiri Khan	19 Apr 2019	OP729854
	SP01RB	Ratchaburi	24 Nov 2018	OP729855
	SP01RB2	Ratchaburi	24 Nov 2018	OP729849
	SP01TK	Tak	26 Dec 2018	OP729851
*A.gilliesiana* (Braasch, 1990)	SP02CR	Chiang Rai	6 May 2019	OP729846
	SP02CR2	Chiang Rai	6 May 2019	OP729845
	SP02CR3	Chiang Rai	6 May 2019	OP729843
	SP02CR4	Chiang Rai	7 May 2019	OP729847
	SP02CR5	Chiang Rai	7 May 2019	OP729844
*A.rainulfiana* (Braasch, 1990)	SP03KN	Kanchanaburi	31 Jan 2019	OP729836
	SP03NT	Nakhon Si Thammarat	2 July 2016	OP729834
	SP03NW	Narathiwat	22 Apr 2018	OP729838
	SP03PC	Phetchaburi	24 Feb 2019	OP729837
	SP03RN	Ranong	20 Apr 2018	OP729833
	SP03RB	Ratchaburi	24 Nov 2018	OP729835
*A.rubromaculata* (You et al., 1981)	SP05CT	Chanthaburi	5 Jun 1028	OP729840
	SP05KN	Kanchanaburi	31 Jan 2019	OP729839
	SP05NA	Nan	5 Dec 2017	OP729842
	SP05RB	Ratchaburi	24 Nov 2018	OP729841

## ﻿Taxonomic accounts

**Family Heptageniidae**

**Genus *Afronurus* Lestage, 1924**

### ﻿ Afronurus cervina

Taxon classificationAnimaliaEphemeropteraHeptageniidae

(Braasch & Soldán, 1984)

7C6012C6-BAA1-5FFF-93A4-054E6E0D8D5F

**Figure 1.**
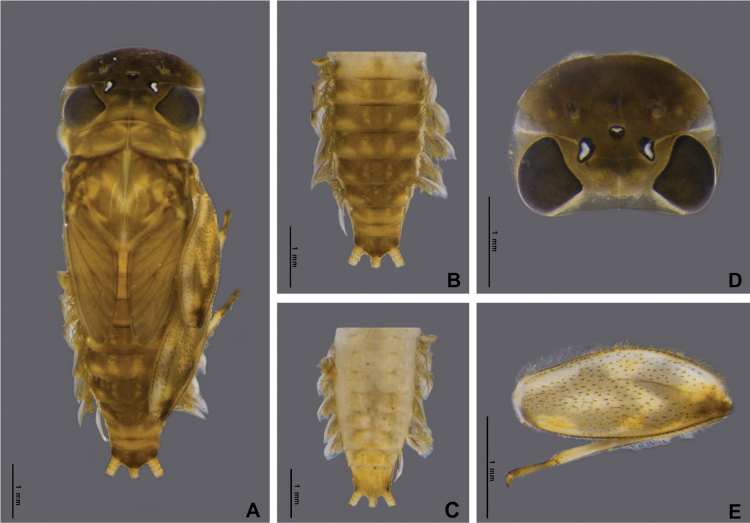
*Afronuruscervina* (Braasch & Soldán, 1984), larval morphology **A** female habitus **B** tergites I–X **C** sternites I–X **D** head **E** hind leg. Scale bars: 1 mm.

**Figure 2.**
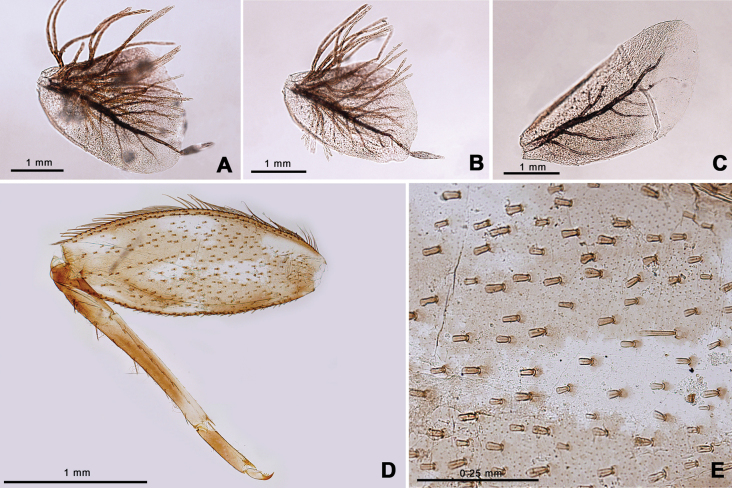
*Afronuruscervina* (Braasch & Soldán, 1984), larval morphology **A** gill V **B** gill VI **C** gill VII **D** hind leg **E** bristles on the dorsal face of the hind femur (middle part). Scale bars: 1 mm (**A–D**); 0.25 mm (**E**).

**Figure 3.**
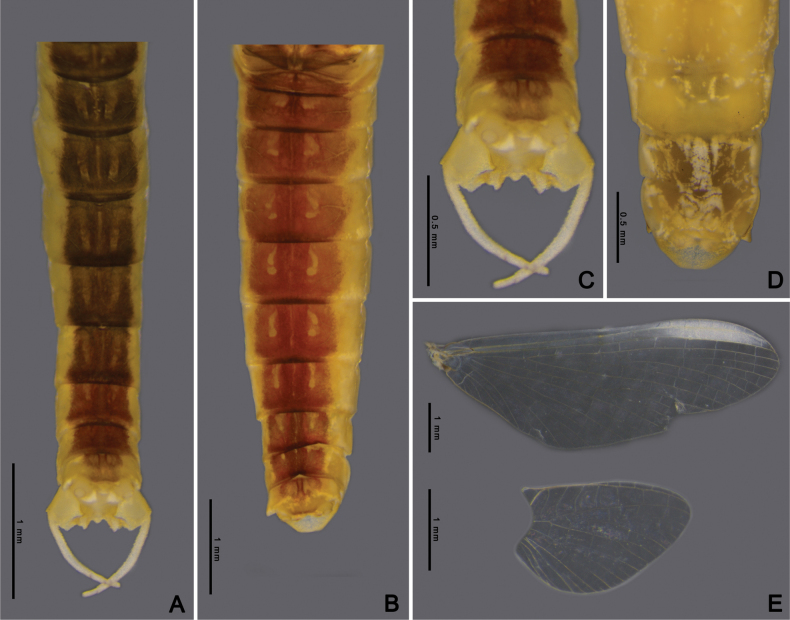
*Afronuruscervina* (Braasch & Soldán, 1984), imaginal morphology **A** male tergites II–X **B** female tergites I–X **C** male genitalia **D** female anal plate **E** fore wing and hind wing. Scale bars: 0.5 mm (**C, D**); 1 mm (**A, B, E**).

**Figure 4.**
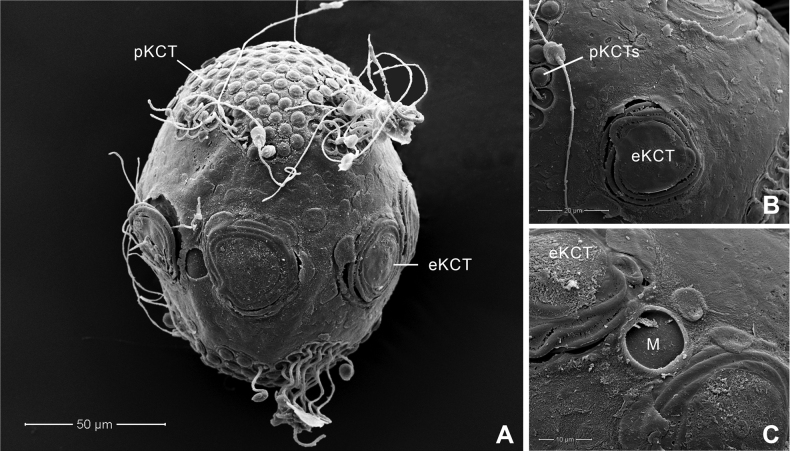
*Afronuruscervina* (Braasch & Soldán, 1984), SEMs of egg morphology **A** general outline of egg **B** chorion surface between polar KCT (pKCT) and equatorial KCT (eKCT) **C** micropyle (M) and enlargement of eKCTs. Scale bars: 50 μm (**A)**; 20 μm (**B**); 10 μm (**C**).

Figs 1A–E , 2A–E , 3A–E , 4A–E , 17A, B

Cinygmina cervina Braasch & Soldán, 1984: 196, figs 14–32, original description (male and female imago, nymph); Venkataraman and Sivaramakrishnan 1989: 118, figs 7, 10 (nymph).
Afronurus cervina – Boonsoong and Braasch 2013: 85.

#### Material examined.

13 nymphs, Chanthaburi Prov., Rattanaburi resort, 12°31'39.9216"N, 102°10'38.9064"E 41 m, 4.V.2023, B. Boonsoong leg. (ZMKU); 5 nymphs, Kanchanaburi Prov., Huai Pilok, 14°39'52.7"N, 98°33'00.3"E, 132 m, 31.I.2019, W. Anuntaya leg. (ZMKU); 4 nymphs, Kanchanaburi Prov., Ban Tao Tan, 14°38'58.199"N, 98°34'55.8006"E, 166 m, 31.I.2019, W. Anuntaya leg. (ZMKU); 7 nymphs, Tak Prov., Oum Yom, 16°48'15.7"N, 99°00'08.3"E, 249 m, 26.XII.2018, W. Anuntaya leg. (ZMKU); 24 nymphs, Ratchaburi Prov., Bor Klueng, 13°31'27.3612"N, 99°14'39.3606"E, 180 m, 24.XI.2018, W. Anuntaya leg. (ZMKU); 9 nymphs, Loei Prov., Nam Thob, 17°15'36.50"N, 101°34'52.90"E, 376 m, 17.XII.2018, W. Anuntaya leg. (ZMKU); 17 nymphs, Ratchaburi Prov., Kang Som Maew, 13°24'22.32"N, 99°6'43.74"E, 207 m, 24.XI.2018, W. Anuntaya leg. (ZMKU); 6 nymphs, Phetchaburi Prov., Huai Sat Yai, 12°38'13.5"N, 99°30'59.34"E, 162 m, 25.II.2018, W. Anuntaya leg. (ZMKU); 7 nymphs, Prachuap Khiri Khan Prov., Huai Sam Rong, 12°3'49.68"N, 99°37'38.76"E, 103m, 25.II.2018, W. Anuntaya leg. (ZMKU).

#### Description.

**Nymph.** See Braasch and Soldán (1984: 196–197, 199, figs 17–32, original description).

**Adult.** See Braasch and Soldán (1984: 196–197, 199, figs 14–16, original description), Braasch (1990: 8).

#### Diagnosis.

Nymph of *Afronuruscervina* (Fig. 1A) has unique characteristics such as a brown abdomen with pale mark and tergites III-IX with two pairs of longitudinal marking on median and lateral. Markings fused on tergites VIII–IX whereas tergite X is dark (Fig. 1B), and no marking on sternites (Fig. 1C). In addition, no marking on anterior margin of head (Fig. 1D). Gill V (Fig. 2A) and gill VI (Fig. 2B) obliquely rounded, triangular, with small projection, and gill VII (Fig. 2C) broad, asymmetrically oval. Marking pattern of hind femur as Figs 1E, 2D. Bristles on the dorsal face of the hind femur are blunt in shape (Fig. 2E).

Adult of *Afronuruscervina* can be distinguished from other *Afronurus* species by its abdominal patterns, dark brown in a band down the middle and yellow along the margin, tergites III–VIII with a pair of thick stripes on the submedian, all tergites with a longitudinal median dark band (Fig. 3A, B). Genital plate emarginated, penis lobes enlarged with terminal edge jagged, between each lobe with cone shaped tubercle (Fig. 3C). The titillators are short, canine tooth-like (Fig. 17A, B). Subanal plate of female adult tongue-shaped and slightly truncated at tip (Fig. 3D). Fore and hind wing as Fig. 3E.

#### Eggs.

Chorionic surface of the egg with large KCTs (knob-terminated coiled threads) or equatorial KCT (eKCT) and small KCTs or polar KCT (pKCT) (Fig. 4A). Both poles were covered with dense pKCTs. Equatorial areas were smooth (Fig. 4B) surrounded with eKCTs and micropyle (M) between eKCTs (Fig. 4C).

#### Distribution.

Chanthaburi, Kanchanaburi, Loei, Phetchaburi, Prachuap Khiri Khan, Ratchaburi and Tak provinces (Fig. 18).

#### Remarks.

*Afronuruscervina* was found for the first time in Ho Chi Min, Vietnam (Braasch and Soldán 1984), then reported in Ban Nam Tok (Chiang Rai province) by Braasch (1990). In this study, we found *A.cervina* at several localities along the northern to southern regions of Thailand. *Afronuruscervina* was found underneath the cobble substrate in slow running water, but can live in wide range of habitats, such as disturbed areas (as in Nakhon Nayok province) and head water streams (as in Loei province). The optimal altitude is between 24 to 527 meters. The abdominal pattern of nymph of *A.cervina* is quite similar to *A.palawanensis* (Braasch and Freitag 2008), but it can be distinguished by the markings on the anterior area of the head (Braasch and Soldán 1984).

### ﻿ Afronurus gilliesiana

Taxon classificationAnimaliaEphemeropteraHeptageniidae

(Braasch, 1990)

54D8775D-7CAD-59B0-8DB7-250B0454CFDF

**Figure 5.**
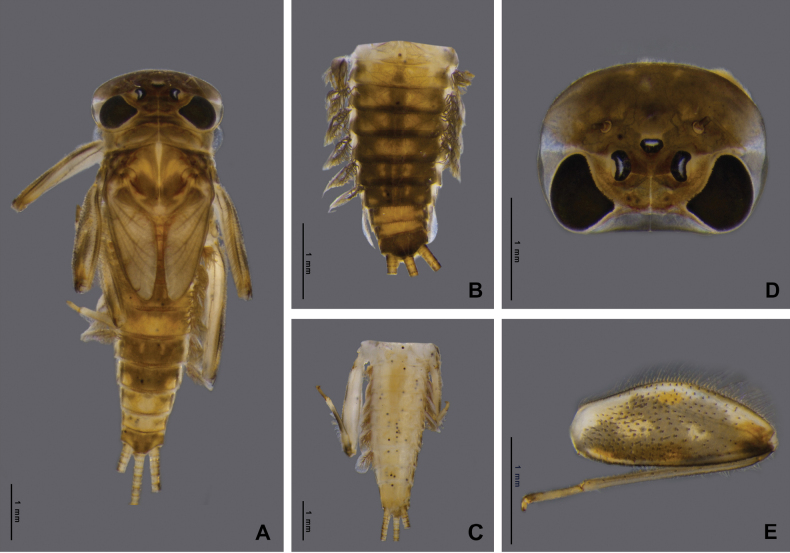
*Afronurusgilliesiana* (Braasch, 1990), larval morphology **A** female habitus **B** tergites I–X **C** sternites VI–X **D** head **E** hind leg. Scale bars: 1 mm.

**Figure 6.**
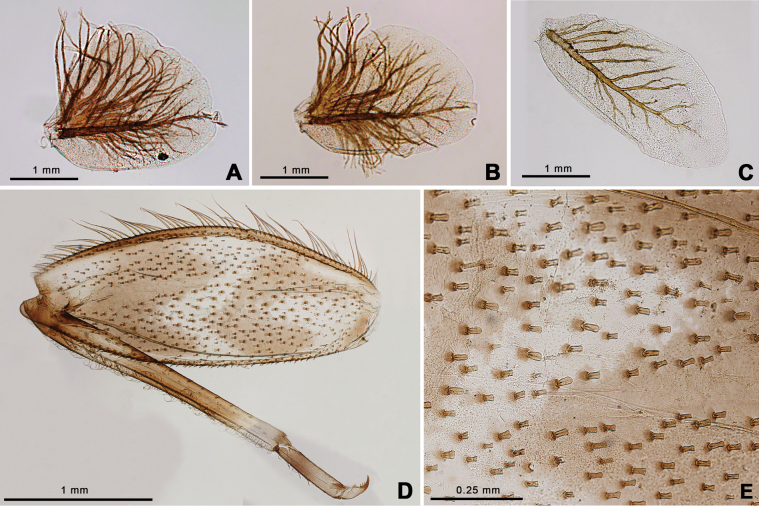
*Afronurusgilliesiana* (Braasch, 1990), larval morphology **A** gill V **B** gill VI **C** gill VII **D** hind leg **E** bristles on the dorsal face of the hind femur (middle part). Scale bars: 1 mm (**A–D**); 0.25 mm (**E**).

**Figure 7.**
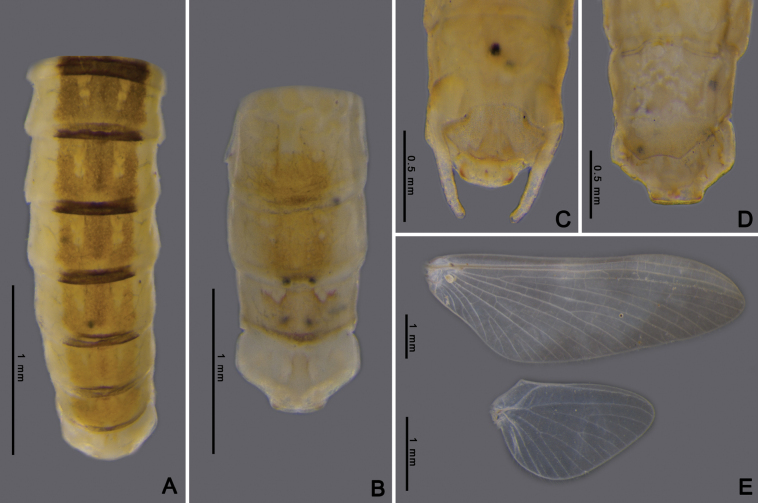
*Afronurusgilliesiana* (Braasch, 1990), imaginal morphology **A** male tergites IV–X **B** female tergites VII–X **C** male genitalia **D** female anal plate **E** fore wing and hind wing. Scale bars: 0.5 mm (**C, D**); 1 mm (**A, B, E**).

**Figure 8.**
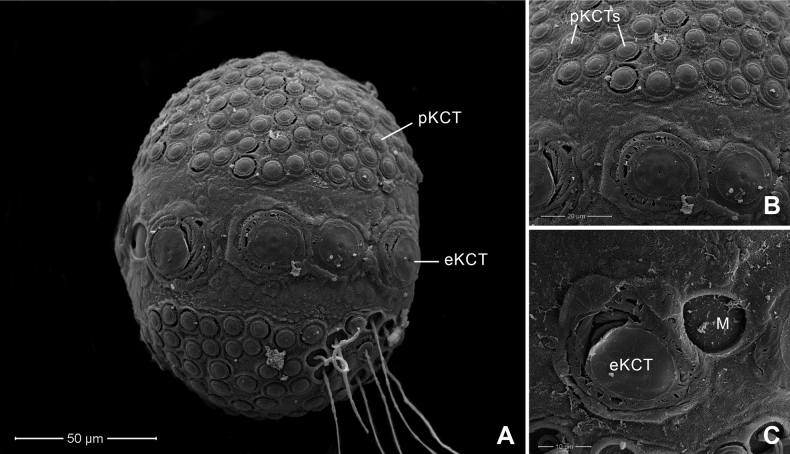
*Afronurusgilliesiana* (Braasch, 1990), SEMs of egg morphology **A** general outline of egg **B** chorion surface between polar KCT (pKCT) and equatorial KCT (eKCT) **C** micropyle (M) and enlargement of eKCTs. Scale bars: 50 μm (**A**); 20 μm (**B**); 10 μm (**C**).

Figs 5A–E , 6A–E , 7A–C , 8A–E , 17C, D

Cinygmina gilliesiana Braasch, 1990: 8, figs 13–16, original description (nymph).
Afronurus gilliesiana – Boonsoong and Braasch 2013: 86.

#### Material examined.

5 nymphs, Chiang Rai Prov., Khun Korn waterfall, 19°51'46.10"N, 99°39'4.70"E, 534 m, 6.V.2019, W. Anuntaya leg. (ZMKU); 4 nymphs, Chiang Rai Prov., Nang Lae Nai Waterfall, 20°3'9.50"N, 99°49'16.90"E, 529 m, 6.V.2019, W. Anuntaya leg. (ZMKU); 3 larvae Chiang Rai Prov., Pong Phrabat Waterfall, 20°0'41.80"N, 99°48'15.10"E, 470 m, 7.V.2019, W. Anuntaya leg. (ZMKU).

#### Description.

**Nymph.** See Braasch (1990: 8, 10, figs 13.1–13.4, 14–16, original description).

**Adult. Male subimago** (in alcohol, Fig. 7A, C, E). ***Head*.** Eyes rounded, blackish on both dorsal and ventral parts, pale laterally. ***Thorax*.** Yellowish with triangular brown patch on sub-median of mesonotum. Forelegs brownish; lengths of femur, tibia, and tarsi 1.64 mm, 1.24 mm, and 0.63 mm, respectively. Midlegs brownish; lengths of femur, tibia, and tarsi 1.68 mm, 1.38 mm, and 0.72 mm, respectively. Hindlegs brownish; lengths of femur, tibia, and tarsi 1.7 mm, 1.42 mm, and 1.22 mm, respectively. Wings transparent; forewing C, Sc and RA thick and yellowish brown, other veins thinner, Sc and RA parallel along the wing, convergent at base, RS and MP forked basally, MA forked at the middle, and CuP and CuA adjacent at base; hindwings rounded, RA and MA adjacent at base of wing, MA and MP forked at the middle (Fig. 7E). ***Abdomen*.** Middle area brown with one pair of longitudinal yellow marks, outer margin pale yellow (Fig. 7A). ***Genitalia***: penis bilobate, expanding laterally to the enlarged lobes, the inner part of lobes with a small cleft (Fig. 17C). Titillators very short, canine-like (Fig. 17C, D), forceps 4-segmented, segment I very small, length ratio of segment II to segment III to segment IV is 0.29: 0.12: 0.1 (Fig. 7C).

**Female subimago** (in alcohol, Figs 7B, D). ***Head*.** Eyes rounded with brownish dorsal part and ventral part dark brown. ***Thorax*.** Yellowish with brown patch at margin. Midlegs brownish; lengths of femur, tibia, and tarsi 1.89 mm, 1.46 mm, and 0.66 mm, respectively. Hindlegs brownish; lengths of femur, tibia, and tarsi 2.3 mm, 1.49 mm, and 0.57 mm, respectively. Wings as in male imagos. ***Abdomen*.** Tergites VII–IX, middle area pale brown with one pair of pale marks on anterior margin, tergite X pale yellow (Fig. 7B). Subanal plate trapezium-shaped and concave at tip (Fig. 7D), length 0.2 mm, width 0.5 mm.

#### Egg.

Chorionic surface covered with pKCTs and eKCTs. Both poles densely covered with pKCTs. Equatorial and subequatorial areas with eKCTs and micropyle beside eKCTs (Fig. 8C); area between pKCTs and eKCTs with indistinct small tubercles (Fig. 8B), areas of two poles of pKTCs ~ 0.7× the size of the whole egg (Fig. 8A).

#### Remarks.

Nymph of *Afronurusgilliesiana* is distinguishable from other species by gill shape, particularly oval-elongated gill I as well as by two large round femoral markings (Figs 5E, 6D). Anterior margin of head with four weak markings (Fig. 5D). Abdomen with marking as shown in Fig. 5A, tergites VIII and IX with pale markings (Fig. 5B) and sternites without marks (Fig. 5C). Gill V (Fig. 6A) and gill VI (Fig. 6B) obliquely rounded, triangular, with small projection; gill VII (Fig. 6C) broad and asymmetrically oval. Bristles on dorsal face of hind femur spatulate in shape (Fig. E).

Adult male can be distinguished by its genitalia: penis bilobate, expanding into laterally enlarged lobes, the inner part of lobes with a small cleft (Fig. 17C).

#### Habitat.

The nymph of *Afronurusgilliesiana* was reported by Braasch (1990) from Mae Sot district, Tak province in northern Thailand. In this study, *A.gilliesiana* was found restricted to three localities in Chiang Rai province. The habitats are unique with high mountain areas, waterfalls, base rock, and some areas of cobbles. The altitude is higher than 400 meters. The nymphs were found attached to the cobbles, away from the base rock with strong water falling from the waterfall. The male and female adults and eggs are described for the first time.

#### Distribution.

Chiang Rai province (Fig. 18).

### ﻿ Afronurus rainulfiana

Taxon classificationAnimaliaEphemeropteraHeptageniidae

(Braasch, 1990)

B8B9F57D-7CC2-5586-8349-9E9BD0A96A4B

**Figure 9.**
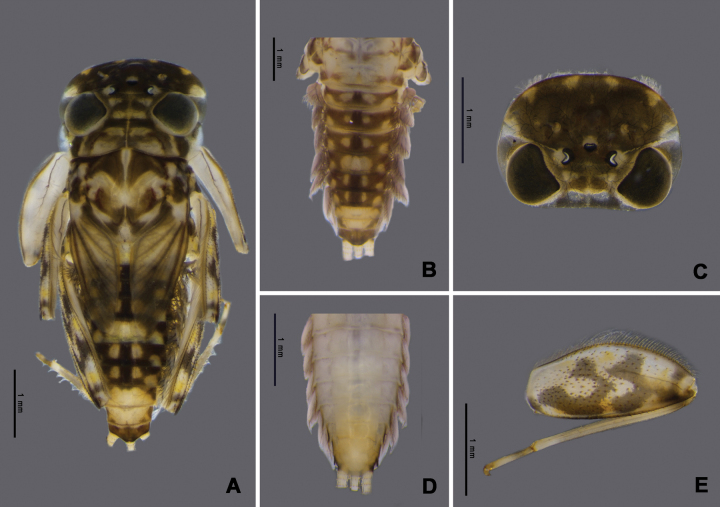
*Afronurusrainulfiana* (Braasch, 1990), larval morphology **A** female habitus **B** tergites I–X **D** sternites III–X **C** head **E** hind leg. Scale bars: 1 mm.

**Figure 10.**
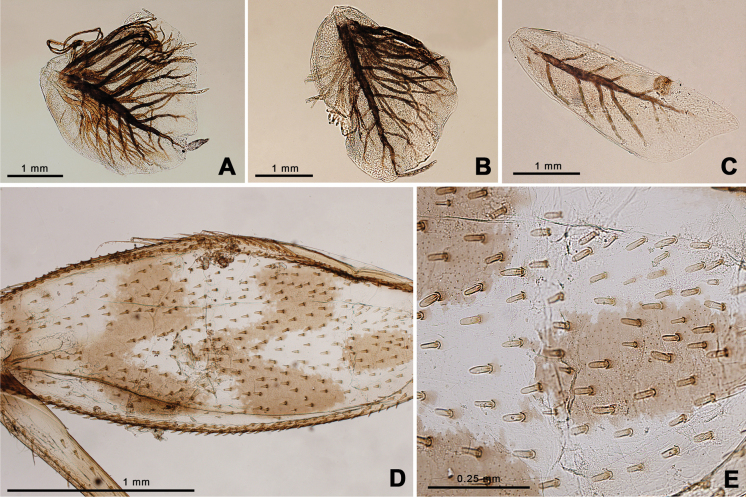
*Afronurusrainulfiana* (Braasch, 1990), larval morphology **A** gill V **B** gill VI **C** gill VII **D** bristles on the dorsal face of the hind femur **E** enlargement of bristles on basal part. Scale bars: 1 mm (**A–D**); 0.25 mm (**E**).

**Figure 11.**
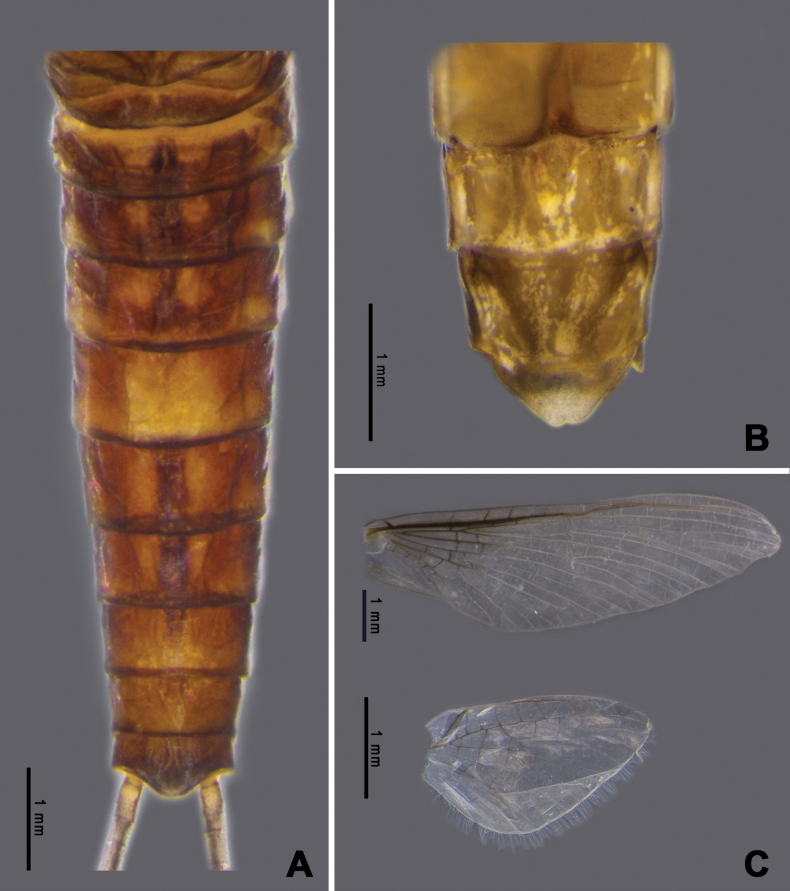
*Afronurusrainulfiana* (Braasch, 1990), female imaginal morphology **A** tergites I–X **B** anal plate **C** fore wing and hind wing. Scale bars: 1 mm.

**Figure 12.**
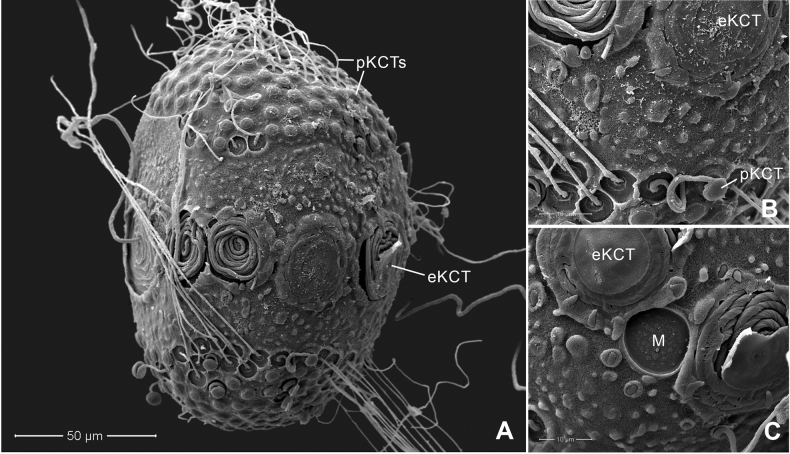
*Afronurusrainulfiana* (Braasch, 1990), SEMs of egg morphology **A** general outline of egg **B** chorion surface between polar KCT (pKCT) and equatorial KCT (eKCT) **C** micropyle (M) and enlargement of eKCTs. Scale bars: 50 μm (**A**); 10 μm (**B**); 10 μm (**C**).

Figs 9A–E , 10A–E , 11A–C , 12A–C

Cinygmina rainulfiana Braasch, 1990: 8, figs 9–12, original description (male and female imago, nymph).
Afronurus rainulfiana – Boonsoong and Braasch 2013: 87.

#### Material examined.

3 nymphs, Thailand, Kanchanaburi Prov., Huai Kha Yeng, 14°36'20.98"N, 98°34'39.8"E, 937 m, 31.I.2019, W. Anuntaya leg. (ZMKU); 12 nymphs, Narathiwat Prov., Klong Aika Ding, 5°47'45.8988"N, 101°50'5.4996"E, 56 m, 22.IV.2018, W. Anuntaya leg. (ZMKU); 1 nymph, Phetchaburi Prov., Huai Mae Kamoei, 12°58'41.4984"N, 99°34'55.401"E, 119 m, 24.II.2019, W. Anuntaya leg. (ZMKU); 4 nymphs, Ranong Prov., Klong Phon Rang, 9°53'39.4002"N, 98°38'28.8996"E, 10 m, 20.IV.2018, B. Boonsoong leg. (ZMKU); 13 nymphs, Ratchaburi Prov., Bo Klueng, 13°31'27.3612"N, 99°14'39.3606"E, 180 m, 24.XI.2018, W. Anuntaya leg. (ZMKU).

#### Description.

**Nymph.** See Braasch (1990: 8, figs 9–12, 18.1–18.3).

**Adult. Female imago** (in alcohol, Fig. 11). ***Head*** brown with black round eyes. ***Thorax*.** Mesonotum brown with pale mark at the middle area, anterior part with dark brown heart-shaped marks. Wings transparent; forewing base area of vein thick and dark brown, RS and MP forked basally, MA forked at the middle; hindwings asymmetrical, 1.6× longer than width, RA and MA adjacent at basal of wing (Fig. 11C). ***Abdomen*.** Dorsally brown marked with yellow, tergites II-V with single pair of circular sub-median marks and another pair of circular posterolateral marks; on tergite V the sub-median mark is fused to form a large square mark, tergites VI and VII with one pair of longitudinal marks each and tergite X pale on anterior part (Fig. 11A); subgenital plate concave at tip; subanal plate extending as triangle-shaped projection and emarginate at tip (Fig. 11B).

#### Egg.

Chorionic surface of egg with dense pKCTs on each pole and eKCTs (Fig. 12A). Equatorial and subequatorial areas with eKCTs and micropyle beside eKCTs (Fig. 12C); area between pKCTs and eKCTs with distinct small tubercles (Fig. 12B), areas of two poles of pKTCs ~ 0.5× that of the whole egg (Fig. 12A).

#### Remarks.

Nymph of *Afronurusrainulfiana* is distinguishable from congeners by the combination of the following characteristics: anterior margin of head with four distinct pale spots (Fig. 9C), lateral surface of the eyes with a large bright triangular spot, and area between eyes with two pairs of circular marks, each mark linked with one straight line. Abdomen tergites II-VII with two pairs of circular markings on sub-median and posterolateral areas (Fig. 9A), pair on sub-median areas fused on tergite V, all markings combined in tergites VIII and IX, and tergite X brown without markings (Fig. 9B). Sternites without markings (Fig. 9C). Gill V (Fig. 10A) and gill VI (Fig. 10B) obliquely rounded, triangular, with apical projection, gill VII narrowly lanceolate (Fig. 10C). Pattern of hind femur as shown in Figs 9E, 10D. Bristles on the dorsal face of the hind femur both blunt and pointed (Fig. 10E). The imago can also be distinguished by the pattern on its abdomen (Fig. 11A).

#### Habitat.

*Afronurusrainulfiana* was described only as a nymph by Braasch (1990) from Saraburi province and then subsequently by Boonsoong and Braasch (2013) in Mae Sot Distinct, Tak province. In this study, *A.rainulfiana* was found in 20 localities in six provinces. The nymphs attach to the undersides of the cobbles submerged in running water. We reported the female characteristics. The male adults of *A.rainulfiana* are still unknown.

#### Distribution.

Kanchanaburi, Narathiwat, Phetchaburi, Ranong, Saraburi and Tak provinces (Fig. 18).

### ﻿ Afronurus rubromaculata

Taxon classificationAnimaliaEphemeropteraHeptageniidae

You, Wu, Gui & Hsu, 1981

190689CC-2CED-5654-97FA-8A29118745A7

**Figure 13.**
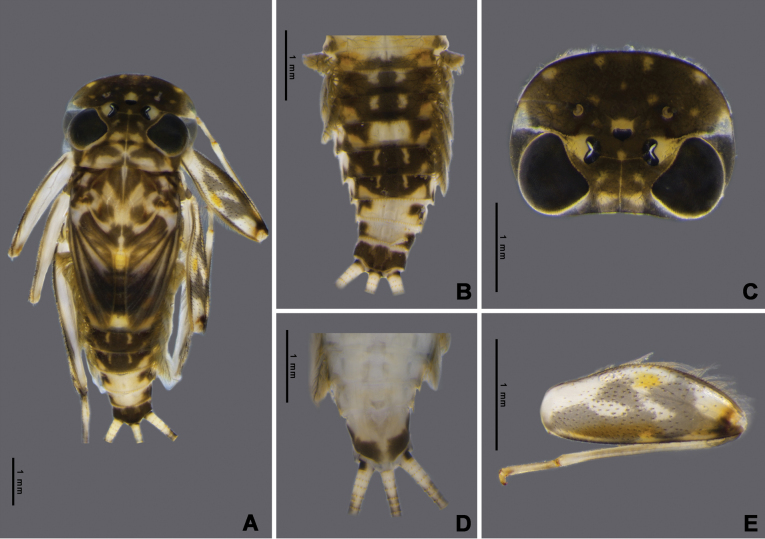
*Afronurusrubromaculata* (You et al., 1981), larval morphology **A** female habitus **B** tergite I–X **D** sternite VI–X **C** head **E** hind leg. Scale bars: 1 mm.

**Figure 14.**
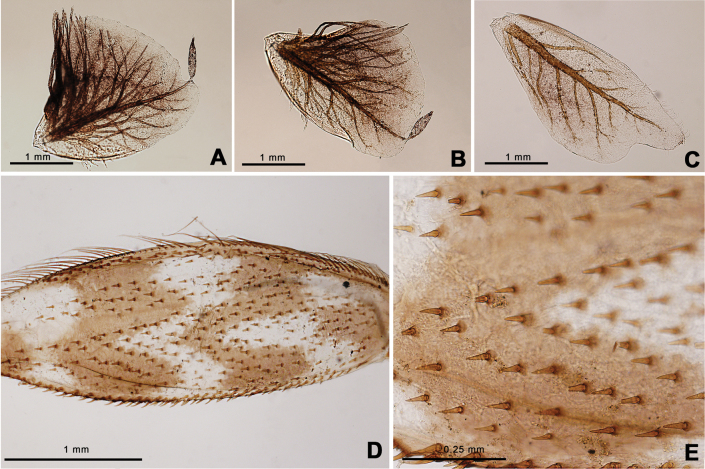
*Afronurusrubromaculata* (You et al., 1981), larval morphology **A** gill V **B** gill VI **C** gill VII **D** bristles on the dorsal face of the hind femur **E** enlargement of bristles on basal part. Scale bars: 1 mm (**A–D**); 0.25 mm (**E**).

**Figure 15.**
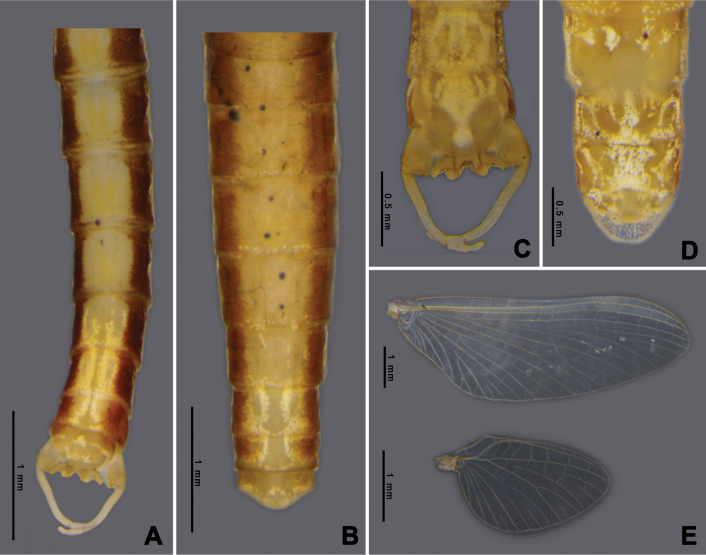
*Afronurusrubromaculata* (You et al., 1981), imaginal morphology **A** male tergites III–X **B** female tergites II–X **C** male genitalia **D** female anal plate **E** fore wing and hind wing. Scale bars: 0.5 mm (**C, D**); 1 mm (**A, B, E**).

**Figure 16.**
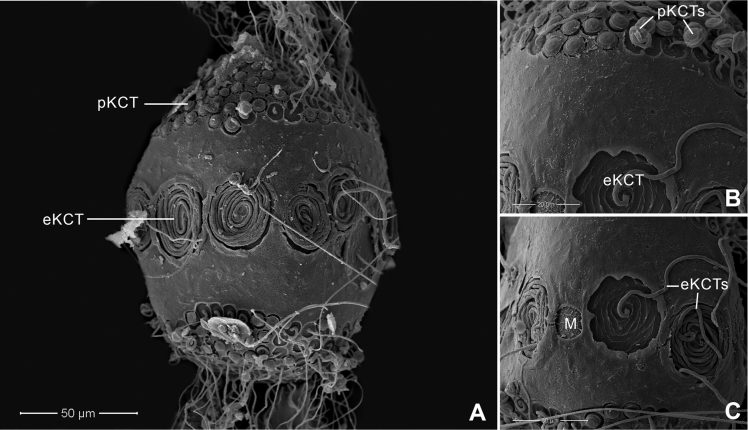
*Afronurusrubromaculata* (You et al. 1981), SEMs of egg morphology **A** general outline of egg **B** chorion surface between polar KCT (pKCT) and equatorial KCT (eKCT) **C** micropyle (M) and enlargement of eKCTs. Scale bars: 50 μm (**A**); 20 μm (**B**); 10 μm (**C**).

**Figure 17.**
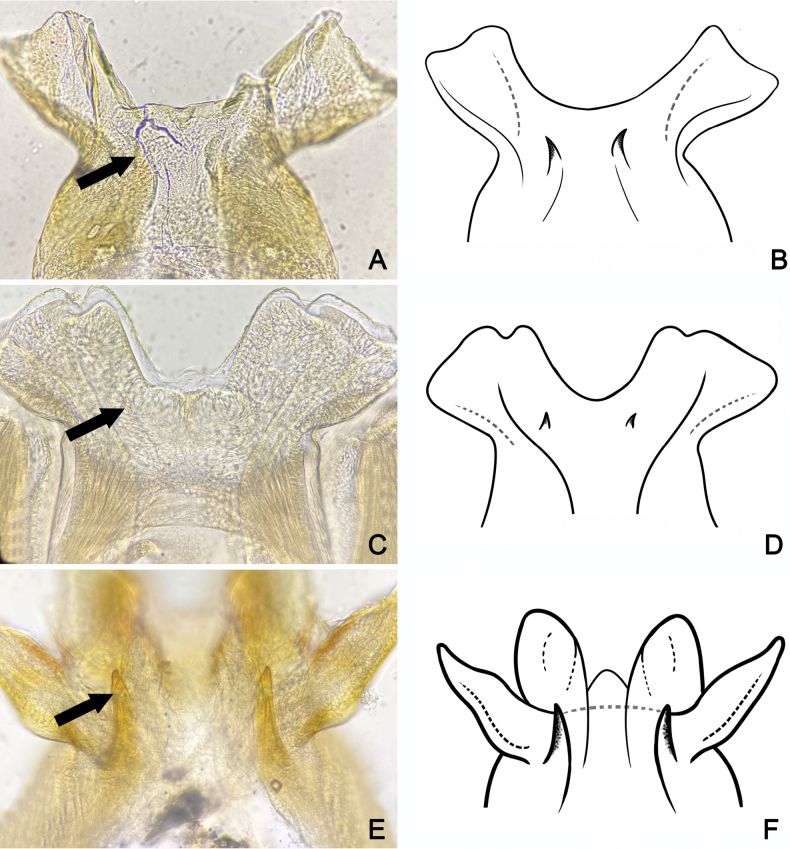
male genitalia **A, B***A.cervina***C, D***A.gilliesiana***E, F***A.rubromaculata* (arrow indicates titillators).

Figs 13A–E , 14A–E , 15A–C , 16A–E , 17E, F

Cinygmina rubromaculata You, Wu, Gui & Hsu, 1981: 4, figs 14–24 (original description, male and female).
Cinygmina rubromaculata – Wu, Chen, Cong & You, 1986: 1, 67.
Cinygmina rubromaculata – Zhou and Zheng 2003: 758, figs 7, 8, 13,17 (nymph first description).
Afronurus rubromaculatus – Braasch and Jacobus 2011: 65.
Afronurus rubromaculata – Boonsoong and Braasch 2013: 88.
Afronurus rubromaculatus – Zhang et al. 2021: 110.

#### Material examined.

11 nymphs, Chanthaburi Prov., Klong Phlu Lang, 12°43.207'N, 102°23.321'E, 115 m, 5.VI.2018, W. Anuntaya leg. (ZMKU); 2 nymphs, Kanchanaburi Prov., Tao Taan, 14°38'58.199"N, 98°34'55.8006"E, 116 m, 31.I.2019, W. Anuntaya leg. (ZMKU); 3 nymphs, Nan Prov., Na noi, 18°19'22.0002"N, 100°43'14.0016"E, 289 m, 5.XII.2017, B. Boonsoong leg. (ZMKU); 15 nymphs, Ratchaburi Prov., Kang Som Maew, 13°24'22.32"N, 99°6'43.74"E, 207 m, 24.XI.2018, W. Anuntaya leg. (ZMKU).

#### Description.

**Nymph.** See Zhou and Zheng (2003: 757, figs 7, 8, nymph first description).

**Adult. Male imago.** See You et al. (1981: 28, figs 14–24, original description).

#### Eggs.

Chorionic surface of egg with dense pKCTs on each pole and eKCTs (Fig. 16A). Equatorial and subequatorial areas with eKCTs and micropyle next to eKCTs (Fig. 16C); the area between pKCTs and eKCTs smooth (Fig. 16B), areas of two poles of pKTCs ~ 0.47× that of the whole egg (Fig. 16A).

#### Diagnosis.

Nymph of *A.rubromaculata* is easily distinguishable from other *Afronurus* species by the following characteristics: anterior margin of head with four distinct pale yellow markings and a row of four pale dots in front of antenna bases and three pairs of pale markings between eyes (Fig. 13C), thorax with pattern as shown in Fig. 13A. Abdominal tergites II-VII with pair of pale marks on sub-median and posterolateral areas, sub-median marking of tergite V fused, large; tergites VIII and IX each with sub-median pale marking; tergite X with anterior pale area (Fig. 13B). Sternites IX and X brown (Fig. 13D). Gills V (Fig. 14A) and VI (Fig. 14B) obliquely rounded, triangular, with projection; asymmetrical gill VII (Fig. 14C). Markings of hind femur as shown in Figs 13E, 14D. Bristles on the dorsal face of the hind femur pointed (Fig. 14E).

Adult male is distinguishable by genitalia and abdominal pigmentation; genital plate emarginated, divided into two lobes, inner lobe broad. The cleft between lobes U-shaped with a small tubercle (Figs 15C, 17E, F), outer lobe canine-like. The titillators robust, canine-like. Forceps comprising four segments, segment I very short, length ratio of segment II to segment III to segment IV is 0.45: 0.14: 0.13 (Fig. 8C). Adult female with anal plate triangular, slightly truncate at tip (Fig. 15D).

#### Distribution.

**Figure 18.**
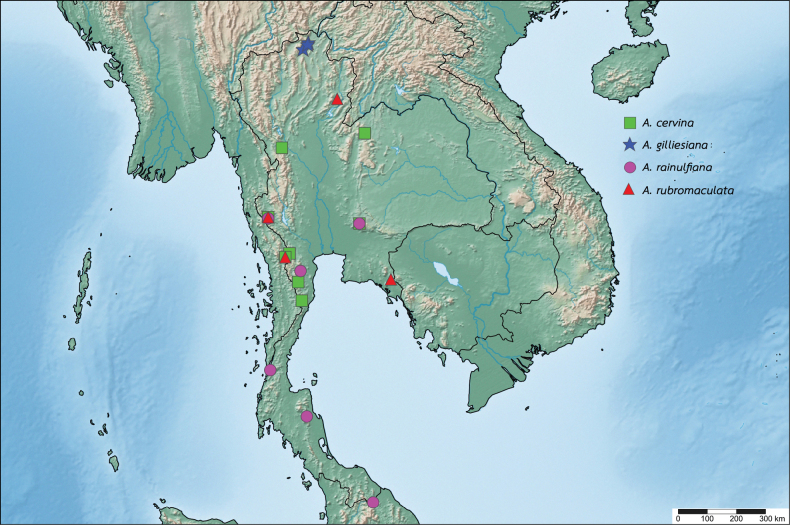
Distribution map of the genus *Afronurus* in Thailand.

Chanthaburi, Kanchanaburi, Nan, and Ratchaburi provinces (Fig. 18).

#### Remarks.

*Afronurusrubromaculata* is a common species in Thai streams and widely distributed (Fig. 18). Suitable localities for *A.rubromaculata* appear to be with cobbles deep in running water, the area mostly covered with riparian fields. *Afronurusrubromaculata* has a unique pattern on the abdomen. She et al. (1995) described the differentiation of the spine position between *A.rubromaculata* and *A.hainanensis*, but Zhou (2013) synonymised *A.rubromaculata* and *A.hainanensis* due to the similarity of the spine on the penes that varies in size. However, in Thailand, the penial character of *A.rubromaculata* is distinct from that of other species of *Afronurus* in Thailand (Table 3). In this study, the egg morphology of *A.rubromaculata* was similar to that of a Chinese specimen, with a smooth surface in the equatorial area (Zhang et al. 2021: fig. 6E).

##### ﻿Molecular analysis

**Figure 19.**
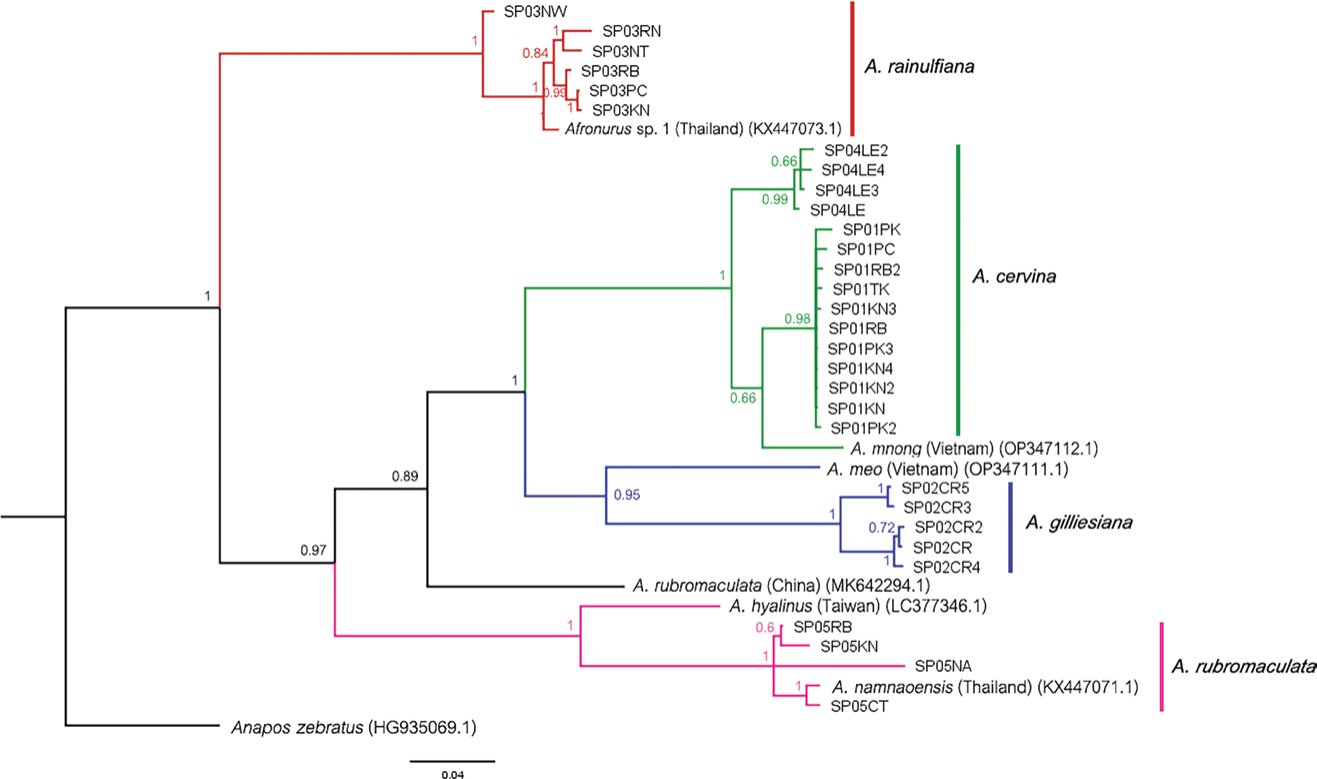
Bayesian inference tree of the DNA barcoding (COI) profile for 37 sequences of the genus *Afronurus* with branch probability support.

The Bayesian inference tree is shown in Fig. 19. The 37 samples of Thai *Afronurus* are grouped into four major clades: *Afronurusrainulfiana*, *A.cervina*, *A.gilliesiana*, and *A.rubromaculata*. Each clade is monophyletic, and strongly supported by the Bayesian posterior probabilities. The intraspecific and interspecific genetic distances are given in Table 2. The range of genetic distances within species is 3%–4%, whereas the range of genetic distances between species is 7%–30%. *Afronurusrainulfiana* is clearly supported as a monophyletic clade with the sequence of *Afronurus* sp.1 (Surat Thani Prov.) from Yanai et al. (2017). The *A.cervina* clade was divided into two sub-clades due to geography; however, the intraspecific genetic distance is 3%. The species *A.mnong* (Vietnam) was clustered with the *A.cervina* clade, with a low genetic distance (7%). By contrast, *A.meo* (Vietnam) was grouped with the *A.gilliesiana* clade and showed a relatively high genetic distance (18%). *Afronurushyalinus* (Taiwan) was clustered with the Thai clade of *A.rubromaculata*. Surprisingly, the *A.rubromaculata* sequence from China is in a different clade than the Thai *A.rubromaculata* sequence (genetic distance 22%), while *A.namnaoensis* was clustered in the *A.rubromaculata* clade with low genetic distance (4%).

**Table 2.** Genetic distances (COI) of seven *Afronurus* species using the Tamura 3-parameter (Gamma).

Species	Tamura 3-parameter (Gamma) distances										
	1	2	3	4	5	6	7	8	9	10	11
1. ***A.cervina* (TH)**	0.03										
2. ***A.gilliesiana* (TH)**	0.23	0.03									
3. *A.hyalinus* (TW)	0.22	0.23	-								
4. *A.meo* (VN)	0.19	0.18	0.24	-							
5. *A.mnong* (VN)	0.07	0.19	0.21	0.18	-						
6. *A.namnaoensis* (TH)	0.23	0.30	0.15	0.26	0.22	-					
7. ***A.rainulfiana* (TH)**	0.22	0.27	0.22	0.24	0.22	0.24	0.03				
8. ***A.rubromaculata* (TH)**	0.22	0.29	0.16	0.26	0.22	0.04	0.24	0.04			
9. *A.rubromaculata* (CN)	0.20	0.23	0.21	0.18	0.19	0.22	0.24	0.22	-		
10. *Afronurus* sp. (TH)	0.22	0.27	0.22	0.26	0.22	0.23	0.02	0.24	0.24	-	
11. *Anaposzebratus* (IT)	0.20	0.26	0.22	0.24	0.21	0.27	0.21	0.25	0.20	0.22	-

CN = China, IT = Italy, TH = Thailand, TW = Taiwan, VN = Vietnam.

### ﻿Key to species of the mature nymph of the genus *Afronurus* in Thailand

**Table:** 

1	Head without any dorsal markings	**2**
–	Head with markings dorsally	**3**
2	Gill I with sharply pointed apex	***A.namnaoensis***
–	Gill I up-turned, banana-shaped	***A.cervina***
3	Head with indistinct spots dorsally	***A.dama***
–	Head with distinct spots dorsally	**4**
4	Head with 2 pairs of pale dots dorsally (Fig. 5D)	***A.gilliesiana***
–	Head with 3 pairs of bright spots dorsally	**5**
5	Gill VII unsymmetrically ovaloid, obtusely pointed apically (Fig. 14C)	***A.rubromaculatus***
–	Gill VII narrowly lanceolate (Fig. 10C)	***A.rainulfiana***

## ﻿Discussion

In this study, four species belonging to the genus *Afronurus* were found in Thailand: *A.cervina*, *A.gilliesiana*, *A.rainulfiana*, and *A.rubromaculata*. The identifications were based on a combination of morphology, ootaxonomy, and molecular analyses. When compared to the previous studies by Braasch (1990) and Boonsoong and Braasch (2013), who reported six species of *Afronurus* in Thailand, two species (*A.dama* and *A.namnaoensis*) of *Afronurus* were not found in this study because the specific habitats of their nymphs were not sampled. *Afronurusdama* was recorded by Braasch (1990) from Nam Tok Ban Du, Chiang Rai province, and only adult specimens were found; Boonsoong and Braasch (2013) referred to the distribution of *A.namnaoensis* from Chaiyaphum, Petchabun, Mae Hong Son, and Chiang Mai provinces, where it was found in high mountain ranges that block dispersion and could be the cause of the absence of this species at our sampling points.

Taken together, the analysis results indicate that *A.rainulfiana* is a common species that is most widely distributed in all regions of Thailand. We found this species to be clearly distinguished from other species based on nymph, imago, and egg morphologies and molecular analysis. The results of molecular analysis showed a more distant relationship between *A.rainulfiana* and other groups, in agreement with the morphological characteristics. For *A.gilliesiana*, the abdominal pattern resembling that of *A.cervina* (Table 3) was also supported by the Bayesian inference tree. In addition, the tree indicated a division of the *A.cervina* clade into two subclades: the first clade includes *A.cervina* from Loei province (northeastern) and the second clade is from the western region of Thailand.

**Table 3.** Comparison of mature nymph characteristics of four Thai *Afronurus* species.

Characters	*A.cervina*	*A.gilliesiana*	*A.rainulfiana*	*A.rubromaculata*
Anterior margin of head	Without any marks^a^	2 pairs of weak marks^b^	3 pairs of bright spots^b^	3 pairs of bright spots
Abdominal pattern	Tergites I, II, VIII, IX pale along all the tergite; tergites III–VII with 2 pairs of pale marks, the pair on sub median exclamation mark-shaped, another pair on sublateral obliquely; tergite V fused; tergite X mostly dark	Tergites I, II, VIII, IX yellowish; tergites III–VII with 2 pairs of markings; 1 pair of elliptical marks on submedian and large circular mark on posterolateral area; tergite V all mark fused; tergite VII with circle marked on posterolateral; tergite X brownish	Tergites I, VIII, IX pale from median to lateral; tergites II-VII with 2 pair of circular marks on sub median and posterolateral areas; tergite V pair of marks on sub median are fused; tergite X brown with no marking	Tergites I, VIII, IX pale from median to lateral areas; tergites III–VII with 1 pair of small longitudinal marks on sub median and another pair of larger marks on posterior area; tergite V with pair of marks on sub median fused; tergite X with transverse marking on anterior area
Setae on hind femur*	B (blunted)	B (spatulated)	B (blunted)	B (blunted)
	M (blunted)	M (spatulated)	M (pointed and blunted)	M (pointed)
	D (blunted)	D (spatulated)	D (pointed)	D (pointed)
Gill VII	Leaflet, asymmetrical, expanded at tip	Leaflet, asymmetrical, 2× longer than wide	Long, end of one side of the gill expanded and pointed at tip	Long, end of gill with 2 lobes; one lobe expanded and rounded at apex
Distribution	Southeast Asia (Thailand, Vietnam)	Southeast Asia (Thailand)	Southeast Asia (Thailand)	Southeast Asia (Thailand), East Palearctic (China)

^a^ Braasch and Soldán (1984); ^b^Braasch (1990; definition based on fig. 21, p. 199); *B = basal area of femur; M = median area of femur; D = distal area of femur.

The intraspecific distances of the Thai *Afronurus* species are low (ranging from 2.8 to 4%), which is lower than the cut-off of 4% (Hebert et al. 2003; Ball et al. 2005; Zhou et al. 2010). The genetic distance data obtained in this study indicates a distance of the different species between 22-30% for the Thai *Afronurus* species. The molecular result was useful for establishing that *A.rainulfiana* was clearly monophyletic but on the contrary, the fact that Thai *A.rubromaculata* clusters with *A.hyalinus* and *A.namnaoensis* but is separated from *A.rubromaculata* from China still needs further study.

Egg characteristics have also proved useful to identify Thai *Afronurus* species (Table 4), as the morphology of the eggs in African and Asian species indicated differences in the size of the large equatorial KCTs (Belfiore et al. 2003). Kang and Yang (1994) also reported differences in equatorial and subequatorial areas of the chorion from Taiwan species.

**Table 4.** Comparison of adult and egg characteristics of four Thai *Afronurus* species.

Characters	*A.cervina*	*A.gilliesiana*	*A.rainulfiana*	*A.rubromaculata*
Abdominal pattern	Middle area brown with yellow patch along the margin, tergites III- VIII with 1 pair thick lines, each tergite with straight line	Middle area brown with 1 pair of longitudinal yellow marks, outer margin pale yellow	Dorsum brown with yellow marks; tergites II-V with 1 pair of circular marks on sub median and another pair of circular marks on posterolateral; on tergite V the mark on sub median is fused to large square shape; tergites VI, VII with 1 pair of longitudinal marks; tergite VIII, IX mostly pale; tergite X pale on anterior part only	Middle area pale yellow with a pair of oval yellow marked, outer margin brownish
Genitalia	Emarginated to forked lobes, the outer ends stronger than the inner, between each lobe cone-shaped tubercle	Bilobed, expanded laterally into enlarged lobes, terminal lobe with 3 serrations, cleft between lobe U-shaped	N/A	Emarginate, each plate divided into 2 lobes, inner lobe broad, outer lobe canine-like, cleft between lobes U-shaped with one serration^c^
Terminal segment of female	Subanal plate tongue-shaped, slightly truncate at tip^a^	Subanal plate extended as trapezium shape, concave at tip	Subgenital plate concave	Subanal plate extended, rounded at tip
			Subanal plate extended as triangle shape and emarginated at tip	
Chorionic surface	Smooth	Smooth	Scattered small tubercles	Smooth
Polar KTCs covering area	0.54×	0.7×	0.5×	0.47×

^a^ Braasch and Soldán (1984), ^b^You et al. (1981: definition based on fig. 19, p. 4).

## Supplementary Material

XML Treatment for Afronurus cervina

XML Treatment for Afronurus gilliesiana

XML Treatment for Afronurus rainulfiana

XML Treatment for Afronurus rubromaculata

## ﻿Additional information

### Author ORCIDs

Anuntaya Wongyam https://orcid.org/0009-0007-9794-564X

Michel Sartori http://orcid.org/0000-0003-3397-3397

Boonsatien Boonsoong https://orcid.org/0000-0002-8166-0021

### Data availability

All of the data that support the findings of this study are available in the main text.
